# Abscisic Acid Enhances Ex Vitro Acclimatization Performance in Hop (*Humulus lupulus* L.)

**DOI:** 10.3390/ijms26146923

**Published:** 2025-07-18

**Authors:** Luciana Di Sario, David Navarro-Payá, María F. Zubillaga, José Tomás Matus, Patricia A. Boeri, Gastón A. Pizzio

**Affiliations:** 1 Sede Atlántica, Universidad Nacional de Río Negro (UNRN), Viedma CP8500, Río Negro, Argentina; ldisario@unrn.edu.ar (L.D.S.); mzubillaga@unrn.edu.ar (M.F.Z.); 2 Sede Viedma, Centro de Investigaciones y Transferencia de Río Negro, Universidad Nacional de Río Negro-Consejo Nacional de Investigaciones Científicas y Técnicas (CIT RIO NEGRO, UNRN-CONICET), Viedma CP8500, Río Negro, Argentina; 3 Institute for Integrative Systems Biology (I2SysBio), Centro Superior de Investigaciones Científicas (CSIC)—University of Valencia, 46908 Paterna, Valencia, Spain; david.navarro.paya@gmail.com (D.N.-P.); tomas.matus@uv.es (J.T.M.)

**Keywords:** PYRABACTIN RESISTANCE 1 LIKE ABA (PYR/PYL) gene family, abiotic stress, abscisic acid (ABA) perception and signaling, in vitro propagation, rustication

## Abstract

*Humulus lupulus* L. (hop) is a multipurpose crop valued for its essential role in beer production and for its bioactive compounds with recognized medicinal properties. Otherwise, climate change represents a major challenge to agriculture, particularly impacting the cultivation of crops with stenoecious characteristics, such as hop. This highlights the urgent need to enhance crop resilience to adverse environmental conditions. The phytohormone abscisic acid (ABA) is a key regulator of plant responses to abiotic stress, yet the ABA signaling pathway remains poorly characterized in hop. Harnessing the publicly available hop genomics resources, we identified eight members of the PYRABACTIN RESISTANCE 1 LIKE ABA receptor family (HlPYLs). Phylogenetic and gene structure analyses classified these HlPYLs into the three canonical ABA receptor subfamilies. Furthermore, all eight HlPYLs are likely functional, as suggested by the protein sequence visual analysis. Expression profiling indicates that ABA perception in hop is primarily mediated by the HlPYL1-like and HlPYL8-like subfamilies, while the HlPYL4-like group appears to play a more limited role. Structure modeling and topology predictions of HlPYL1b and HlPYL2 provided insights into their potential functional mechanisms. To assess the physiological relevance of ABA signaling in hop, we evaluated the impact of exogenous ABA application during the ex vitro acclimatization phase. ABA-treated plants exhibited more robust growth, reduced stress symptoms, and improved acclimatization success. These effects were associated with reduced leaf transpiration and enhanced stomatal closure, consistent with ABA-mediated drought tolerance mechanisms. Altogether, this study provides the first comprehensive characterization of ABA receptor components in hop and demonstrates the practical utility of ABA in improving plant performance under ex vitro conditions. These findings lay the groundwork for further functional studies and highlight ABA signaling as a promising target for enhancing stress resilience in hop, with broader implications for sustainable agriculture in the face of climate change.

## 1. Introduction

*Humulus lupulus* L. (hop) is a well-known crop in the brewing industry, valued for its contribution of key bittering and flavoring components such as bitter acids, flavonoids, and essential oils. Beyond its industrial use, hop is also classified as a medicinal plant due to its rich array of bioactive compounds with significant therapeutic potential, such as xanthohumol (XN) and 8-prenylningenin (8-PN), which have been widely studied for their health-promoting properties [1]. The hop tissue with commercial value is the inflorescence of female plants (cones), which contains a large amount of glandular trichomes loaded with a yellow resin called lupulin [1]. Lupulin accounts for 10–30% of the cone dry weight, and the most important flavoring agent is the bitterness of alpha and beta acids, known collectively as bitter acids.

Hop plants production by seeds or vegetative propagation (rhizomes and cuttings) may result in strong disadvantages, such as male plants propagules, poor phytosanitary quality, and accumulations of pathogens that reduce productivity. In vitro propagation of hops represents an effective alternative [2,3]. For instance, in vitro plant regeneration from meristems produces high phytosanitary quality propagules, which prevent and control the dissemination of pathogens in hop cultivation. Additionally, in vitro propagation of nodal segments ensures female plant production with low genetic variability. Nevertheless, the efficiency of the acclimatization phase, during which plants adapt to harsh ex vitro conditions, plays a critical role in the success of micropropagation protocols. During this process, it is crucial to carefully address stomatal dysfunctions and high transpiration rates. The phytohormone abscisic acid (ABA) plays a key role in inducing drought tolerance through stomatal closure, induction of antioxidant capacity, and synthesis of osmoprotectant solutes [4,5].

ABA triggers stomatal closure [5], regulates the hydraulic response to prolonged drought [6], controls carbohydrate metabolism during recovery from stress [7], and modulates salt perception and posterior exclusion when plants are under salinity stress [8,9]. Additionally, this phytohormone regulates the secondary metabolism to enhance plant acclimatization to abiotic stress, for instance, by increasing the content of different terpenes and anthocyanins with antioxidant properties [10,11]. Moreover, ABA also induces the production of osmocompatible solutes, such as proline, betaines, galactinol, and sugars [12,13].

The ABA signal transduction pathway is then initiated by the hormone perception in target cells by the family of PYRABACTIN RESISTANCE1 LIKE (PYL) receptors [4,14]. ABA perception induces the formation of a ternary complex between ABA, PYL receptors, and clade A protein phosphatase type 2C (PP2Cs) [15,16,17]. Consequently, the inhibition of the subclass III SNF1-related protein kinases 2 (SnRK2s) by PP2Cs is abolished [18,19]. ABA-activated SnRK2s turn on the downstream signaling through different effectors, from transmembrane channels to transcription factors [4]. In a second level of regulation, the activity and half-life of the core ABA signaling components are regulated by accessory kinases, the proteasome system pathway, and the circadian system [20,21,22,23].

Climate change brings significant challenges to agriculture and food production, impacting crops that thrive in regions with specific environmental requirements, i.e., those with stenoecious characteristics, such as *Humulus lupulus*. Therefore, it is imperative to examine the ability of plants to withstand challenging environmental conditions. Plant response to abiotic stress heavily relies on ABA signal transduction, while the ABA receptor family has been methodically identified in a wide range of crops, such as quinoa [24,25], grape [26], wheat [27], palm [28], and benthamiana [29], among other species. To discover innovative strategies to cope with environmental stress and improve hop production, this study aims to (i) identify and characterize the PYL receptor gene family in *Humulus lupulus*, and (ii) evaluate the role of ABA in regulating leaf transpiration and enhancing ex vitro acclimatization performance. We hypothesize that ABA signaling, mediated by PYL receptors, contributes to physiological adjustments that improve plant adaptation during acclimatization.

Taking advantage of the public hope genomics resource [30], we have identified eight members of HlPYL ABA receptors, implementing the genome-search method. To categorize this, HlPYL phylogenetic and gene structure analyses were conducted. The result grouped HlPYLs in the three classical subfamilies of ABA receptors: PYL1-like, PYL4-like, and PYL8-like. Moreover, the expression pattern of HlPYLs at the tissue-specific level showed that PYL1-like and PYL8-like receptors are predominantly expressed and should lead to ABA perception across hop plants. By contrast, PYL4-like receptors should have residual functions. Additionally, to evaluate the potential of each HlPYL to be functional, conserved domains analyses and visual examinations of protein sequence alignment were performed. In addition, the protein structure of HlPYL1b and 2 was modeled by homology, and topology analysis was performed to visualize relevant distinctive residues. Finally, we have evaluated the role of ABA in regulating stomatal closure, leaf transpiration, and plant ex vitro acclimatization, resulting in reduced water loss, reduced plant stress symptoms, and increased growth. As a result, both water loss and stress symptoms in plants were reduced, leading to improved growth performance.

## 2. Results

### 2.1. Genome-Wide Identification of PYLs in H. lupulus

**Figure 1:**
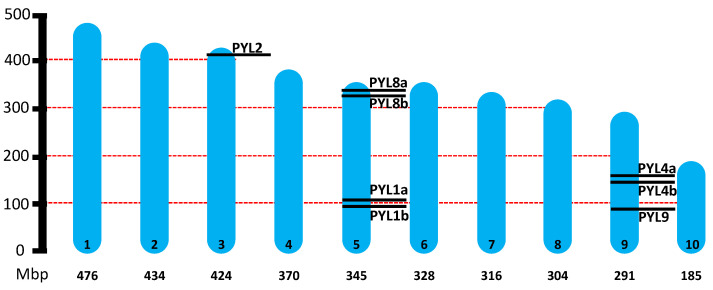
Chromosomal location of eight HlPYL genes across the *Humulus lupulus* genome. The ten chromosomes (numbered inside) of hop are shown in scale based on their physical size. Under each chromosome, the estimated size in Mbp: Mega base pair.

The hop genome is approximately 2.8 Gb in size, distributed across 10 chromosomes (2n = 18 + XY) [30]. A total of eight *PYL* genes were identified in the hop genome (Table 1). The open reading frame (ORF) lengths of the *HlPYL* genes ranged from 558 bp to 669 bp, with an average of 582 bp, while the encoded proteins varied in length from 185 to 222 amino acids, averaging 193 residues. Chromosomal mapping revealed that these eight *HlPYL* genes are unevenly distributed across only three out of ten hop chromosomes (Figure 1). Notably, *HlPYL2* is located on the sexual chromosome 3. Besides, chromosome 5 harbors the highest number of *PYL* genes, containing four *HlPYLs*, while chromosome 9 contains three. In contrast, chromosomes 1, 2, 4, 6, 7, and 10 contain no *HlPYL* genes (Figure 1).

### 2.2. Phylogeny, Gene Structure Analysis, and Tissue-Specific Expression Pattern of HlPYLs

**Figure 2:**
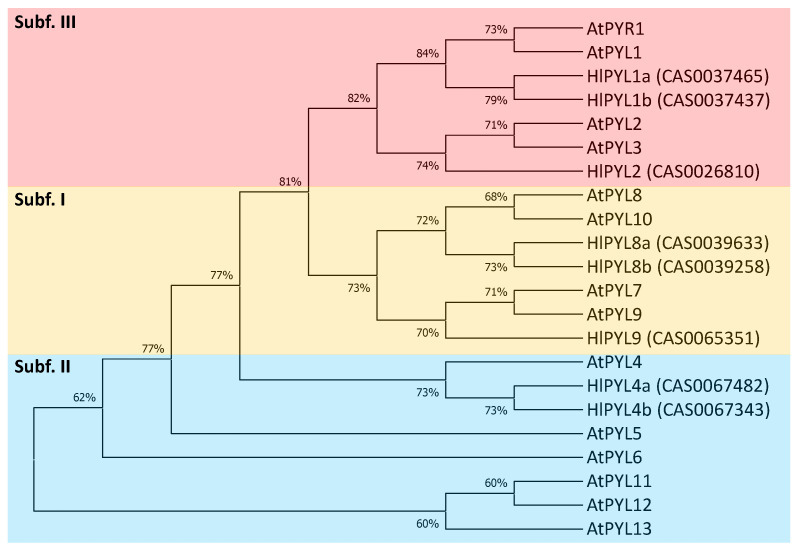
Phylogenetic analysis of the PYL gene family in *Humulus lupulus* L. and *Arabidopsis thaliana*. The tree was built in MEGAx (software version 10.2.6), using the LG model and maximum-likelihood method (1000 bootstraps). Orange, blue, and red clades represent subfamilies I, II, and III, respectively. The percentage of replicate trees in which the associated taxa clustered together is shown next to the branches.

To investigate the phylogenetic relationships of PYL receptors in *Humulus lupulus*, a maximum-likelihood (ML) tree was constructed using full-length protein sequence alignments of the eight identified HlPYLs and 14 AtPYLs (Figure 2). As there is no standard nomenclature for *PYL* genes in hop, the *HlPYLs* were named based on their phylogenetic relationships with their *Arabidopsis thaliana* counterparts. Consistent with the previous studies in *Arabidopsis* and other species [4,14,24,26,31], HlPYLs clustered into three conserved subfamilies. Specifically, subfamily I included three gene members (*HlPYL8a*, *HlPYL8b*, and *HlPYL9*), subfamily II comprised two members (*HlPYL4a* and *HlPYL4b*), and subfamily III consisted of the remaining three genes (*HlPYL1a*, *HlPYL1b*, and *HlPYL2*).

**Figure 3:**
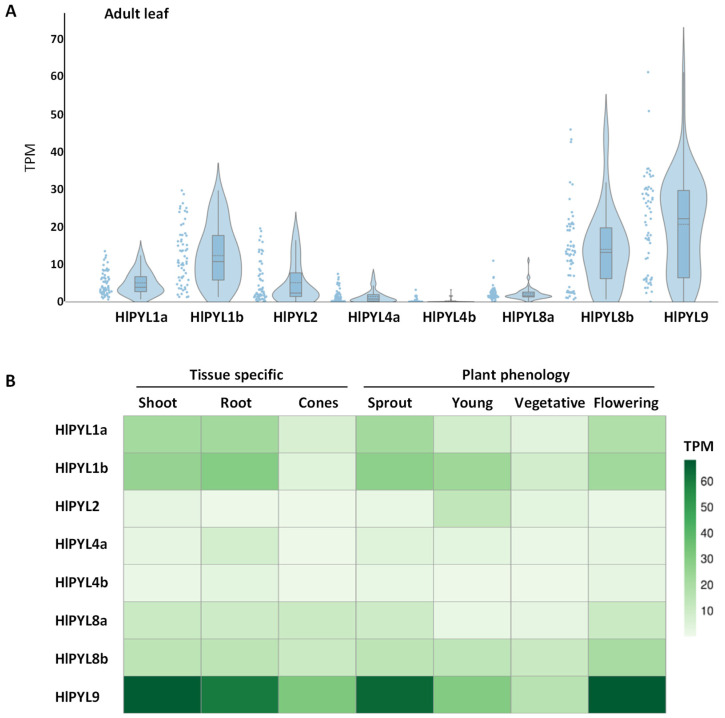
Tissue-specific expression patterns of *HlPYLs*. (**A**) Leaf expression of hop *PYL* genes across public transcriptomic data. RNA-Seq raw data (Illumina; 61 runs) were downloaded from the SRA-NCBI and analyzed. Expression data were normalized to transcripts per kilobase of exon model per million mapped reads (TPM) values. Violin plots show the overall frequency distribution of data points, while the inner box plots mark interquartile ranges, mean (dotted line), and median (solid line) values. (**B**) Mean TPM is visualized in a heatmap of plant phenology and tissue-specific *HlPYLs* expression. Public RNAseq corresponding leaf (Illumina; 61 runs), shoot system (Illumina; 25 runs), root (Illumina; 19 runs), cones (Illumina; 2 runs), sprout (Illumina; 44 runs), young plant (Illumina; 14 runs), vegetative (Illumina; 37 runs) and flowering (Illumina; 25 runs) were treated as in (**A**).

To further support the phylogenetic classification, we analyzed the gene structure of *HlPYLs*, including untranslated regions (UTRs), exons, and introns, which can provide insights into gene family evolution [32] (Supplemental Figure S1). The analysis revealed that five genes were intronless (HlPYL1a, HlPYL1b, HlPYL2, HlPYL4a and HlPYL4b); whereas three genes (HlPYL8a, HlPYL8b and HlPYL9) contain two introns. Notably, genes within the same subfamily shared similar exon–intron structures, further validating the subfamily classifications derived from the phylogenetic analysis (Figure 2). To assess the tissue-specific expression patterns of *HlPYLs*, we analyzed publicly available RNA-seq data across different tissues (adult leaf, shoot, root, and flower cones) and developmental stages (sprout, young plant, vegetative, and flowering) (Figure 3). The results revealed distinct expression profiles among *HlPYL* family members. Members of subfamily I (*HlPYL8b* and *HlPYL9*) and subfamily III (*HlPYL1a* and *HlPYL1b*) were highly expressed across most tissues and stages, suggesting they play a ubiquitous role in ABA perception and signaling. In contrast, subfamily II members (*HlPYL4a* and *HlPYL4b*) exhibited low or residual expression, indicating a potentially limited functional role under the conditions analyzed.

### 2.3. Conserved Motif, Protein Alignments, and 3D Topology Analysis of PYLs in Hop

To confirm the presence of the PYR_PYL_RCAR domain in each HlPYL protein sequence, a motif/domain search was performed using the Pfam and NCBI-CDD databases via the MOTIF search tool. The PYR_PYL_RCAR domain is a hallmark of ABA receptors and is essential for their proper functionality. All eight HlPYL proteins identified in this study exhibited the presence of this conserved domain (Supplemental Figure S2), suggesting that they are likely functional ABA receptors.

**Figure 4:**
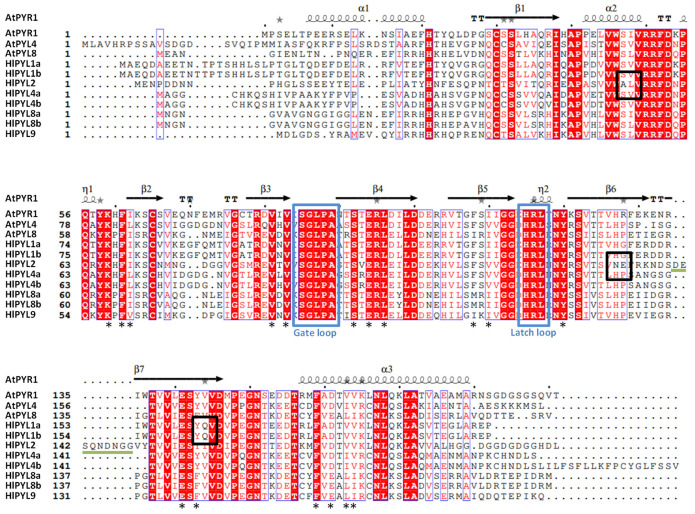
Amino acid sequence alignment of *Arabidopsis* and hop ABA receptors. Sequence and secondary structure alignment of ABA receptors are indicated. The secondary structure is shown according to the crystallographic structure of AtPYR1 (Protein DataBank Code 3k3k; β = beta-sheet; α = alpha-helix). The final visualization was generated using the ESPRIPT v3.0 tool. Blue boxes indicate the position of the gate and latch loops. Black boxes indicate outstanding residue changes from the consensus. Asterisks mark residues involved in interactions with ABA. The green line denotes an extra loop found in HlPYL2.

A close inspection of the amino acid sequence and secondary structure alignment of the AtPYLs and HlPYLs, generated using ESPRIPT (v3.0) [33], shows a high degree of conservation (Figure 4). It is particularly relevant that the conservation of key features involved in ABA binding, such as the gate loop and the latch loop [34]. Besides, a conservation in key residues required for hormone-receptor interaction (Figure 4) is also observed. This fact is in agreement with the idea that the eight HlPYLs are functional, capable of binding ABA and forming the ternary complex with PP2Cs proteins. It is interesting to note that HlPYL2 has a novel extra loop, between B6 and B7 sheets, close to the latch loop (Figure 4).

This loop, in turn, could modulate the ABA perception dynamics and/or the ternary complex ABA-PYL-PP2C formation. Furthermore, inside this loop, we can see a putative phosphorylation target in S142. The function of this extra loop in HlPYL2 remains open to be characterized. Besides, in HlPYL2, we also detect a significant deviation from the consensus sequence in the residue A54 and N132-E133. In the equivalent position of A54, a serine (S) is usually present in other PYLs, a putative phosphorylation target. Regarding N132-E133 (asparagine-glutamic acid) residues, the consensus at these positions is H-P (histidine-proline) and represents a significant change from a positive to a negative charge in this loop. Interestingly, N132-E133 residues are close to the latch loop and to the novel B6-B7 loop (Figure 4), in range to modify ABA binding and/or phosphatases interaction. On the other hand, we also detect a significant change in residues Q162/Q163 in HlPYL1a/b, respectively. The consensus at this position is that the small hydrophobic residue valine (V) and HlPYL1a/b have a big polar glutamine (Q). This residue is also close to the latch loop, and it is next to two key residues involved in ABA direct coordination, Y161/162 and E159/160 (Figure 4).

**Figure 5:**
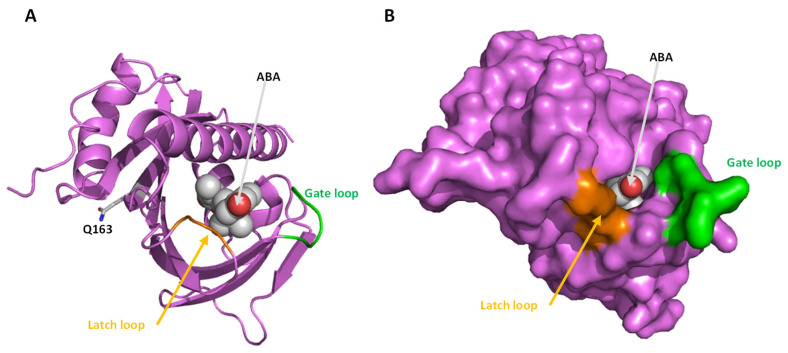
HlPYL1b protein 3D model. Topology was predicted by homology modeling in Swiss-Model using the PDB template 5mob. The model is shown by PyMOL as cartoons (**A**) and as a protein surface (**B**). The model quality estimation by GMQE Global is 0.86, and QMEANDisCo Global: 0.85 ± 0.06.

**Figure 6:**
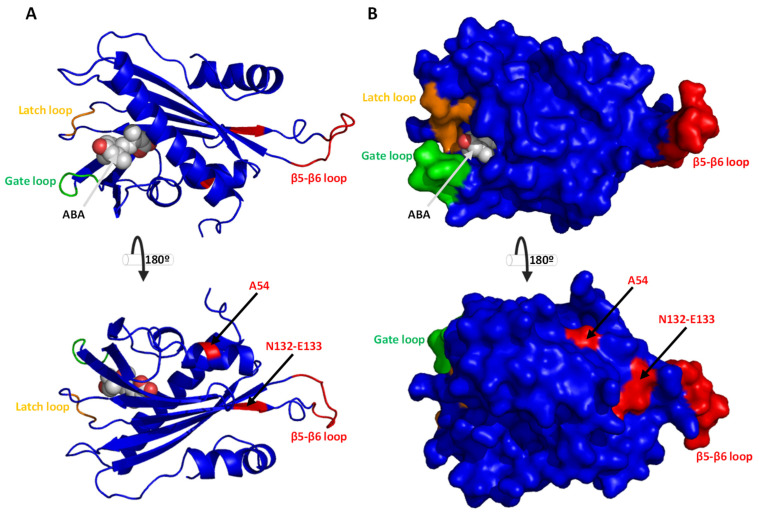
HlPYL2 protein 3D model. Topology was predicted by homology modeling in Swiss-Model using the PDB template 3kdh. The model is shown by PyMOL as cartoons (**A**) and as a protein surface (**B**). The model quality estimation by GMQE Global is 0.81, and QMEANDisCo Global: 0.83 ± 0.05.

To further inspect the new features of HlPYL1 and 2, the protein 3D structure was obtained by homology modeling using the SwissModel in silico tool [35]. The models of HlPYL1b and HlPYL2 were obtained using as templates the topology of SlPYL1 (PDB-ID 5mob) and AtPYL2 (PDB-ID 3kdh), respectively (Figure 5 and Figure 6). Protein structure-estimated quality indicated that models were reliable (Supplemental Figures S3 and S4). In the topology analysis of HlPYL1b, residue Q163 does not face the ABA interaction pocket (Figure 5A). Despite its close proximity to the latch loop and key residues involved in ABA binding, it is unlikely that Q163 disturbs or plays any significant role in hormone coordination. Furthermore, Q163 appears to be buried within the protein structure (Figure 5B), making it inaccessible for modulating homodimer or ternary complex formation. In contrast, the topology analysis of HlPYL2 reveals that residues A54, N132, and E133 are exposed on the protein surface (Figure 6A,B) and are therefore potentially accessible for mediating interactions with other proteins. However, the distance between these residues and the protein binding surface is not within the range necessary to influence interactions with other PYLs or phosphatases. Regarding the novel loop in HlPYL2, located between B6 and B7 sheets, it is positioned on the side opposite to the gate of the ABA binding pocket and is unlikely to play a role in modulating ABA interactions or establishing the ternary complex. Nonetheless, this loop is exposed on the protein surface, and the phosphorylation-targeted residue S142 is accessible for modification by kinases (Figure 6A,B).

### 2.4. ABA Effects in Plant Ex Vitro Acclimatization and Transpiration

**Figure 7:**
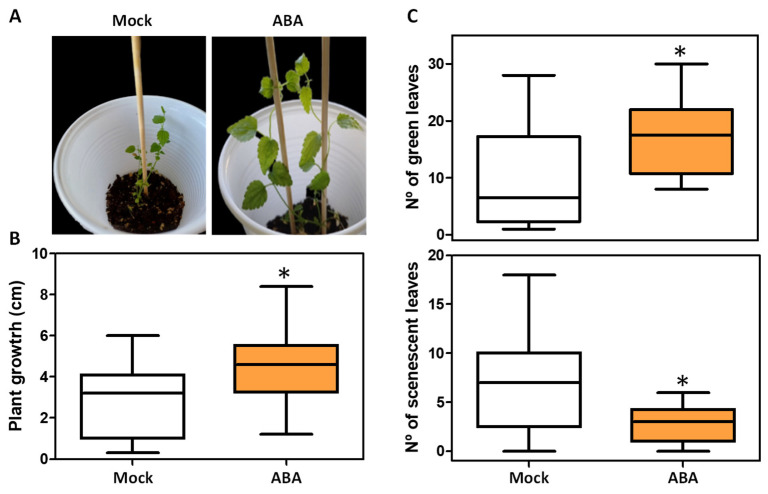
ABA treatment improves in vitro acclimatization and rustication in *Humulus lupulus* L. *cv ‘Mapuche’*. (**A**) A representative mock and 50 µM ABA sprayed plants; (**B**) plant growth quantification; (**C**) Number of green leaves (upper panel) and number of senescent leaves (lower panel). Phenotypes were evaluated after 21 days of ex vitro growth. The asterisk indicates a significant difference with respect to mock (*p* ≤ 0.05).

The acclimatization and rustication of whole plants derived from in vitro culture is a critical step to ensure successful adaptation to the stressful ex vitro environment. Usually, plant survival during this phase is often compromised by excessive water loss and premature senescence. The phytohormone ABA plays a central role in mediating plant responses to stress. However, after 21 days of transplantation into substrate under ex vitro conditions, no significant difference in survival rate was observed between plants treated with 50 µM ABA and mock (approximately 80% survival in mock- and ABA-treated plants). Nevertheless, notable differences were detected in other phenotypic traits (Figure 7). Plants treated with 50 µM ABA showed significantly increased growth compared to mock-treated plants (mean plant height, 4.46 cm vs. 2.73 cm, respectively), a higher number of green leaves (17.07 vs. 9.75) and fewer senescent leaves (2.92 vs. 6.89), indicating improved physiological status and acclimatization under ABA.

**Figure 8:**
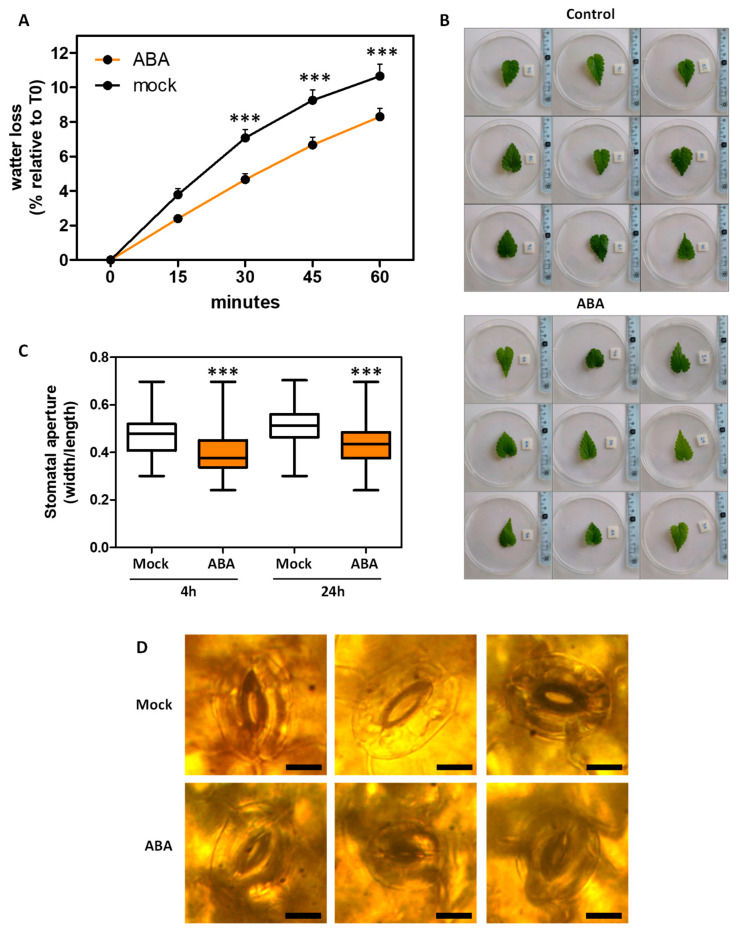
Transpiration and stomatal regulation by ABA in *Humulus lupulus* L. *cv ‘Mapuche’*. (**A**) Loss of fresh weight of mock or 100 µM ABA-treated leaves. Line plots indicate mean ± SE. (**B**) Photograph of representative excised leaves assayed in (**A**). (**C**) ABA-induced stomatal closure in hop leaves at 4 h and 24 h post-treatment. Box plot marks interquartile ranges, mean (line), and maximum and minimum values (error bars). (**D**) Photograph of representative stomata measured at 24 h post mock or 10 µM ABA treatment; scale bars = 200 µm. Student’s *t*-test was performed, and asterisks indicate a significant difference with respect to mock (*** *p* ≤ 0.001).

In several plant species, ABA regulates leaf transpiration by modulating stomatal aperture [5]. However, this process remains uncharacterized in hop. To address this, a water loss assay was performed, revealing that treatment with 100 µM ABA significantly reduced leaf transpiration in hop (Figure 8A,B). Consistently, 10 µM ABA application also induces stomatal closure at both 4 and 24 h post-treatment (Figure 8C,D), supporting a conserved role for ABA in promoting water retention through stomatal regulation.

## 3. Discussion

Abscisic acid (ABA) plays a pivotal role as a plant regulator, influencing various agronomical traits, including stress tolerance, seed maturation and germination, as well as shoots and root growth and development. Moreover, the exogenous application of ABA has been shown to act as a biostimulant, enhancing crop stress resilience [36]. Beyond plant systems, ABA can also be perceived by mammalian cells, where it has been proposed to improve several aspects of human health, including sleep disorders, depression, memory, and pain [37].

ABA is a key hormone in the regulation of physiological responses to abiotic stress [4,14]. Central to ABA signaling is the PYRABACTIN RESISTANCE1-LIKE (PYL) gene family, which encodes the ABA receptors and initiates the signal transduction cascade. The identification and functional characterization of PYL receptors across species has highlighted their potential for improving stress tolerance traits such as drought and salinity resistance in crops [9,12,26,29,38]. These advances are particularly relevant in the context of global climate change, which demands new strategies for improving crop resilience [31,39,40,41,42].

In this study, we identified the ABA receptor gene family in *Humulus lupulus* L., uncovering eight *PYL* genes distributed among the three classical phylogenetic clades: the dimeric PYL1-like subgroup (three members), and the monomeric PYL8-like and PYL4-like subgroups, with three and two members, respectively (Figure 2), accordingly to other reports in different crops [26,28,29,43]. Based on phylogenetic analysis, we propose standardized nomenclature for the eight *HlPYLs* based on their closest *Arabidopsis* orthologs (Table 1). Interestingly, the PYL4-like subfamily is underrepresented, with only two members, a pattern previously reported only in *Citrus sinensis* [43], where this subfamily typically includes more genes. In contrast to *C. sinensis*, the expression of *HlPYL4a* and *HlPYL4b* is notably low across multiple tissues (Figure 3), suggesting a limited functional role. Instead, ABA perception in hop is likely mediated predominantly by members of the PYL1-like and PYL8-like subfamilies. In particular, HlPYL1a, HlPYL1b, HlPYL8b, and HlPYL9 show strong expression in a range of tissues (adult leaves, shoots, roots, and cones) and developmental stages (sprouting, vegetative, and flowering phases) (Figure 3). A similar pattern has been observed in *Phoenix dactylifera*, where PYL8-like receptors play a dominant role in ABA perception [28].

All eight HlPYL proteins contain a conserved PYR_PYL_RCAR domain, suggesting their potential functionality as ABA receptors (Supplemental Figure S2). This is further supported by the high degree of conservation observed in key residues and structural motifs, including the gate and latch loops that are critical for ABA binding (Figure 4). Interestingly, HlPYL2 possesses an additional loop near the latch region, which could, in principle, influence ABA perception dynamics and/or the formation of the ternary ABA–PYL–PP2C complex. However, topology analysis indicates that this extra loop is located on the opposite side of the ABA-binding gate (Figure 6), making a direct role in ABA binding or complex formation unlikely. Nonetheless, the presence of a putative phosphorylation site at residue S142 within this surface-exposed loop raises the possibility of a novel regulatory mechanism, which remains to be experimentally validated. In *Arabidopsis thaliana*, ABA receptor activity is modulated by multiple kinases, such as Target Of Rapamycin (TOR), Arabidopsis Early Flowering 1 (EL1)-like casein kinase (AEL), C-terminally Encoded Peptide Receptor 2 (CEPR2), and Cytosolic ABA Receptor Kinase 1 (CARK1), which fine-tune ABA signaling through phosphorylation [22].

In vitro propagation of hop has been demonstrated as an effective technique to prevent pathogen transmission and ensure the clonal multiplication of female plants [2,3]. A critical step in this process is acclimatization or rustication, during which plantlets must adapt to the harsher ex vitro environment, particularly by tolerating abiotic stresses [3]. ABA plays a central role in regulating drought tolerance [4,5] by promoting stomatal closure, enhancing antioxidant defenses, and stimulating the production of osmoprotectants. In this context, our results showed that ABA treatment in hop induced stomatal closure, significantly reduced leaf transpiration, and improved plant growth performance during ex vitro acclimatization (Figure 7 and Figure 8). These findings are consistent with previous reports demonstrating the positive effects of ABA application on plant acclimatization, such as in *Ulmus minor* [44]. Additionally, ABA may mitigate leaf stress and reduce senescence, thereby contributing to healthier plant development post-transplantation. Together, these observations emphasize the importance of considering post in vitro culture treatments in securing successful plant adaptation to ex vitro conditions.

**Figure 9:**
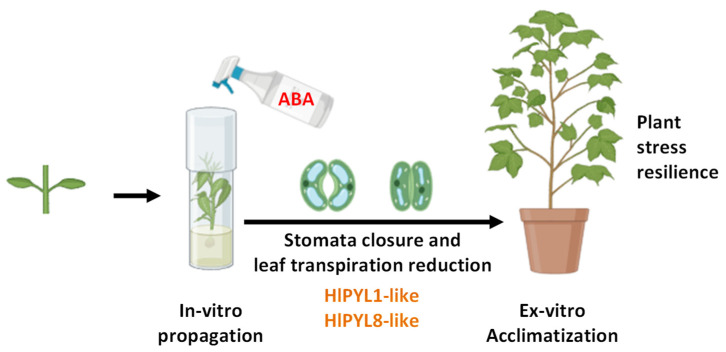
ABA enhances ex vitro acclimatization performance in *Humulus lupulus* L. Hop acclimatization and rustication improvement after ABA treatment in terms of plant growth and stress symptoms. ABA in leaves could be perceived by receptors of dimeric PYL1-like and monomeric PYL8-like sub-families.

In conclusion, ABA perception in *Humulus lupulus* is likely mediated primarily by PYL1-like and PYL8-like receptors, leading to physiological responses such as stomatal closure and reduced leaf transpiration, which enhance ex vitro acclimatization performance (Figure 9). This study lays a foundational framework for future functional analyses of *HlPYL* receptors and highlights the potential of utilizing ABA signaling to improve stress resilience in hop.

## 4. Materials and Methods

### 4.1. Genome-Wide Identification of PYL Genes in Humulus lupulus L.

The *Arabidopsis thaliana* PYL protein sequences were used as a query to perform a BLASTP search against the local protein database of *Humulus lupulus* [30]: AtPYR1 (AT4G17870), AtPYL1 (AT5G46790), AtPYL2 (AT2G26040), AtPYL3 (AT1G73000), AtPYL4 (AT2G38310), AtPYL5 (AT5G05440), AtPYL6 (AT2G40330), AtPYL7 (AT4G01026), AtPYL8 (AT5G53160), AtPYL9 (AT1G01360), AtPYL10 (AT4G27920), AtPYL11 (AT5G45860), AtPYL12 (AT5G45870), AtPYL13 (AT4G18620). Eight unique proteins were identified and considered as candidate hop PYL proteins.

### 4.2. Phylogenetic and Gene Structure Analysis of HlPYLs

The chromosome physical location of the eight *HlPYL* was obtained from the HopBase genome DB; this is the chromosomal coordinate corresponding to each hit after the blast search [30]. A multiple sequence alignment of the eight identified hop proteins and 14 *Arabidopsis* PYLs was performed using ClustalW, followed by phylogenetic tree construction using the maximum-likelihood method (ML), using MEGA X (software version 10.2.6) [45] with a bootstrap value of 1000. The gene UTR–exon–intron structure of these eight *HlPYL* genes was obtained from the HopBase genome DB annotation files.

### 4.3. Analysis of ABA Receptor Expression in Hop Public Transcriptomic Data

Metadata for public RNA-Seq experiments (Illumina platform) assigned to *Humulus lupulus* were obtained from the Sequence Read Archive (SRA)-NCBI. These SRA metadata were automatically classified according to tissue, stage, cultivar, organism, and flower sex using the ontology-based system described in PlantaeViz [46]. The curated metadata are provided in Supplementary File S1, with a total of 61 runs for leaf, 25 runs for shoot system, two runs for cone, and 19 runs for root. These were used to evaluate the expression of ABA receptors across different tissues. Runs were trimmed using fastp and aligned to the cv. “Cascade” hop genome using STAR (default parameters + -runMode alignReads --limitOutSJcollapsed 8,000,000 --limitIObufferSize 220,000,000). The gene models for the cv. “Cascade” hop genome, derived from maker and transdecoder, was obtained from HopBase [30]. Although gene IDs with the “HUMLU_CAS” prefix were given to the combined transdecoder and maker gene set, a single combined GFF was not provided by the database. Therefore, after individual transdecoder and maker gffs were obtained from the database, gene IDs were renamed to the “HUMLU_CAS” structure based on a provided mapping file. A combined GFF was generated with all gene models and their corrected gene IDs, and the file is currently shared in the PlantaeViz platform (https://plantaeviz.tomsbiolab.com/hop/downloads/, accessed on 1 March 2025). Count matrices were obtained using featureCounts (-t “exon” -C -g “featurecounts_id”) using the combined annotation file and summarizing counts at the gene level by using a custom “featurecounts_id”. Multimapping reads were not counted. Expression data were normalized to transcripts per kilobase of exon model per million mapped reads (TPM) values. To visualize the tissue-specific HlPYLs expression, a heat map was constructed using the CLUSTVIS web tool [47].

### 4.4. Conserved Motifs Analysis, Protein Alignments, and Homology Modeling of HlPYLs 3D Structure

The MOTIF search tool [48] was employed to identify the presence of the conserved PYR_PYL_RCAR domain within each HlPYL protein sequence, using both the Pfam and NCBI-CDD databases. This domain includes characteristic features such as the Polyketide cyclase/dehydrase and lipid transport domain, as well as the Bet_v1-like domain. Protein alignments between *Arabidopsis* (*AtPYLs*) and *hop* (*HlPYLs*) proteins were performed with the MegAlign 7.0 software from the DNASTAR Lasergene suite. Alignment graphics, along with the annotated secondary structure of AtPYR1 (Protein DataBank ID 3k3k), were generated using the Easy Sequencing in PostScript (ESPript, v3.0) program [33]. To investigate the protein 3D structure, homology modeling of HlPYL1b and HlPYL2 was carried out using the Swiss-Model interactive workspace [35]. Templates used for modeling were SlPYL1 (5 mob, for HlPYL1b) and AtPYL2 (3 kdh, for HlPYL2). Model quality was assessed using the QMEANDisCo global score function, along with the QMEANDisCo local scores (per residue) and several QMEAN Z-scores, including QMEAN, Cβeta, All Atom, Solvation, and Torsion scores. The QMEAN Z-score provides an estimate of the structural ‘nativeness’, with higher values indicating more reliable models [49,50]. Based on these evaluations, the 3D models of both *HlPYL1b* and *HlPYL2* were considered reliable. Final 3D visualization and structural analysis were performed using Pymol (PyMOL Molecular Graphics System, Version 1.6, Schrödinger, LLC9).

### 4.5. Plant Material, ABA Treatment, and Acclimatization to Ex Vitro Conditions

Uninodal segments of *Humulus lupulus* cv. ‘Mapuche’ derived from in vitro plants were cultured in 225 mL glass jars at 21 ± 2 °C under a long-day photoperiod (16:8 h light:dark) with a light intensity of 50 μmol/m^2^/s provided by white, fluorescent lamps [3]. The culture medium consisted of half-strength Murashige and Skoog basal medium (½ MS), supplemented with vitamins, macro- and micronutrients. The medium was solidified with 0.65% (*w*/*v*) agar (Sigma, St. Louis, MA, USA), supplemented with 3% (*w*/*v*) sucrose, and was adjusted to pH 5.8 prior to autoclaving for 25 min at 121 °C.

Following 21 days of in vitro culture, fully developed seedlings were subjected to an acclimatization process aimed at facilitating their physiological adaptation from the highly controlled in vitro environment to non-sterile ex vitro conditions. This process began 24 h after a foliar application of 50 µM racemic ABA (Phytotech, Lenexa, KS, USA) solution or a mock treatment on both the adaxial and abaxial leaf surfaces, with 15 plants treated under each condition. Subsequently, the plants (*n* = 30) were transferred to 220 mL pots filled with Growmix^®^ substrate (Terrafertil, Moreno, Argentina) and covered with a film to maintain high humidity levels. The plants were incubated at room temperature under long-day photoperiod in the growth room. During the first three weeks, the film was gradually opened and fully removed at the end of this period. At this point, the following parameters were evaluated: plant survival, plant growth, and the number of green and senescent leaves. Then, surviving plants were transplanted into 0.5 L pots and placed in an outdoor environment.

### 4.6. Leaf Transpiration and Stomatal Aperture Assays

Leaves from four-month-old *Humulus lupulus* cv ‘Mapuche’ plants were sprayed with 100 µM racemic ABA (Phytotech, Lenexa, KS, USA) 24 h before sampling and then excised, together with untreated leaves (mock). The water loss kinetic analysis was subsequently measured. Data from nine leaves per treatment were plotted as the percentage of their initial fresh weight at each time point. Individual leaves were weighed every 15 min for three hours using an analytical balance. The experiment was repeated three times.

The stomatal aperture assay was conducted using whole-leaf images from four-month-old *Humulus lupulus* plants. To score ABA-induced stomatal closure, leaves were first incubated for 24 h in stomatal opening buffer containing 10 mM KCl and 10 mM 2-(N-morpholino)-ethanesulfonic acid–KOH, pH 6.2, at 20 °C. Subsequently, the leaves were incubated for 4 or 24 h in fresh stomatal opening buffer, with or without the addition of 10 µM ABA [51]. Stomatal images were taken using a Leica SFL 100 microscope with a Leica MC170 camera, and the aperture of 30 to 40 stomata per leaf (determined by width-to-length ratio) was measured using ImageJ 1.54g software in two independent experiments.

### 4.7. Statistical Analysis

Data were visualized in box plots. The statistical difference between mock and ABA treatments was calculated using a *t*-test or one-way ANOVA followed by Dunnett’s test, and *p*-value indicated with asterisks (* *p* ≤ 0.05; ** *p* ≤ 0.01 and *** *p* ≤ 0.001).

## Figures and Tables

**Table 1:** Basic Information of ABA Receptors in *Humulus lupulus* L.

Gene ID	Gene Name	Genome Location (Start..Stop)	ORF Length (bp)	Protein Size (aa)
HUMLU_CAS0037465	HlPYL1a	CH5/Scaffold 24 (101098846..101099927)	600	200
HUMLU_CAS0037437	HlPYL1b	CH5/Scaffold 24 (97872371..97873468)	603	201
HUMLU_CAS0026810	HlPYL2	CH3/Scaffold 1533 (420945668..420946409)	615	204
HUMLU_CAS0067482	HlPYL4a	CH9/Scaffold 49 (153780561..153781466)	579	192
HUMLU_CAS0067343	HlPYL4b	CH9/Scaffold 49 (147637069..147638078)	633	211
HUMLU_CAS0039633	HlPYL8a	CH5/Scaffold 24 (335968664..335971620)	579	192
HUMLU_CAS0039258	HlPYL8b	CH5/Scaffold 24 (323689689..323692723)	579	192
HUMLU_CAS0065351	HlPYL9	CH9/Scaffold 49 (93847944..93850402)	558	185

These eight unique proteins were identified and considered as candidate hop PYL proteins.

## Data Availability

Data is contained within the article and supplementary material, or publicly available on-line.

## Supplementary Materials

The supporting information can be downloaded at https://www.mdpi.com/article/10.3390/ijms26146923/s1.
