# Supplementary material for: Abscisic Acid Enhances Ex Vitro Acclimatization Performance in Hop (Humulus lupulus L.)
Source: Int J Mol Sci. 2025 Jul 18;26(14):6923. doi: 10.3390/ijms26146923 (PMC12294887; doi:10.3390/ijms26146923)
Supplement: Supplementary file 1 [file ijms-26-06923-s001.zip › Supplementary figures S1 to S4.pdf]

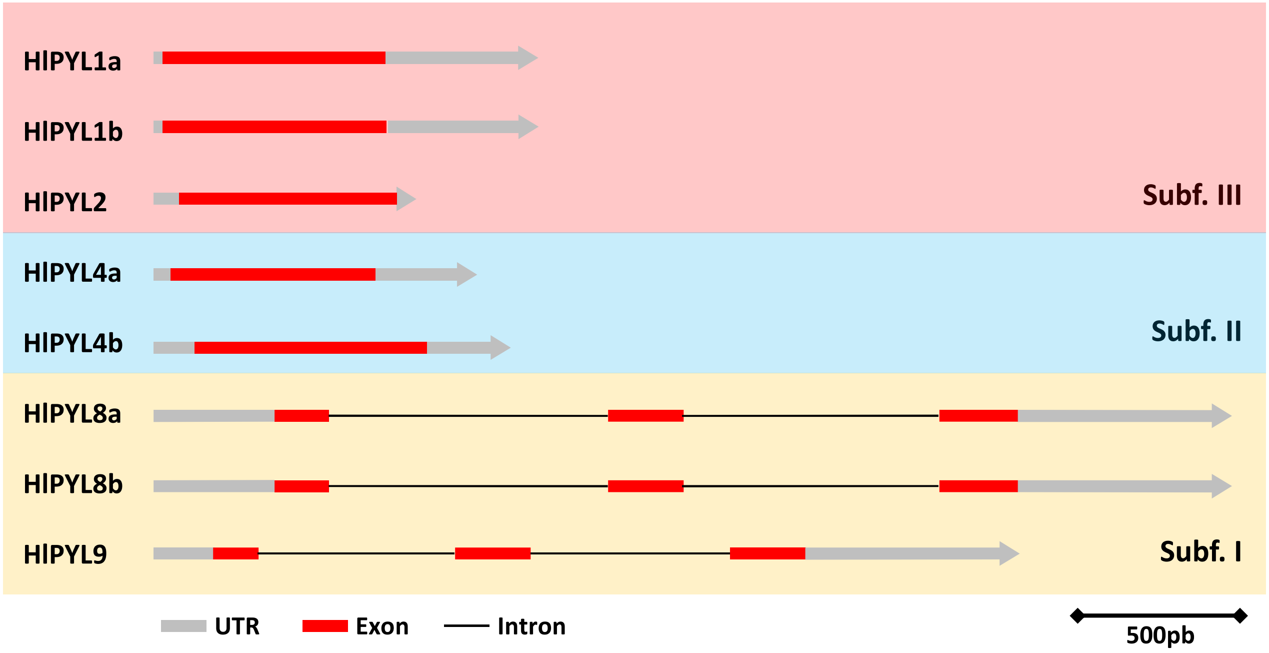

Supplemental figure 1. **Gene structure of PYL genes in *Humulus lupulus L.*** In red exons, in grey UTRs and black lines represent introns. Orange, blue and red clades represent subfamilies I, II and III, respectively.

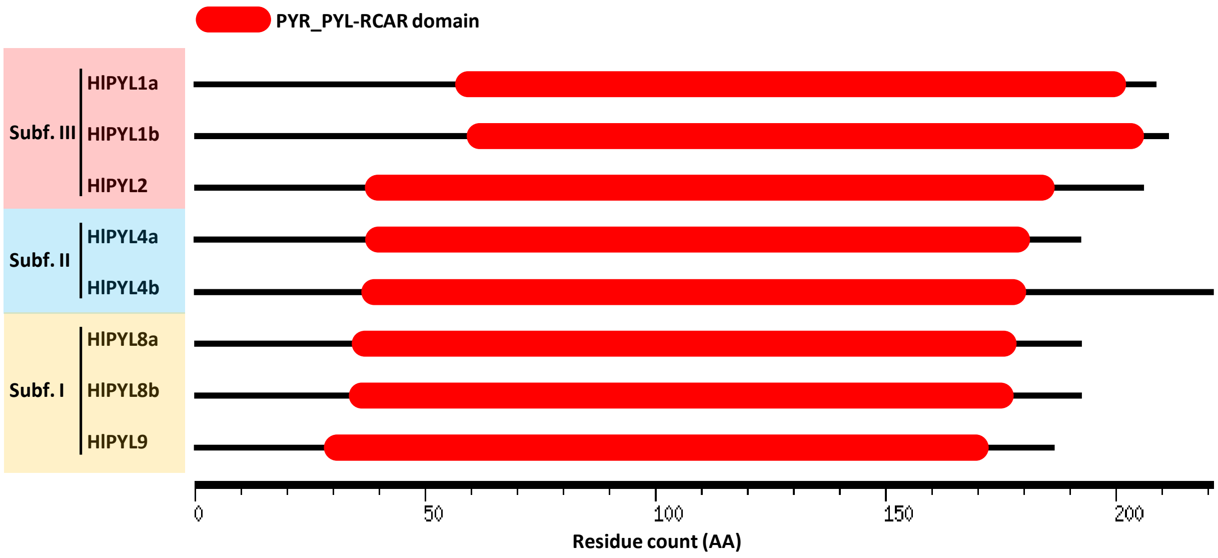

Supplemental figure 2. **Domain analysis of HlPYL proteins.** The motif/domain search was done in pfam and NCBI-CDD databases. In red rectangles the PYR_PYL_RCAR domain (PYR_PYL-RCAR motif includes the Polyketide cyclase and the Bet_v1-like domain).

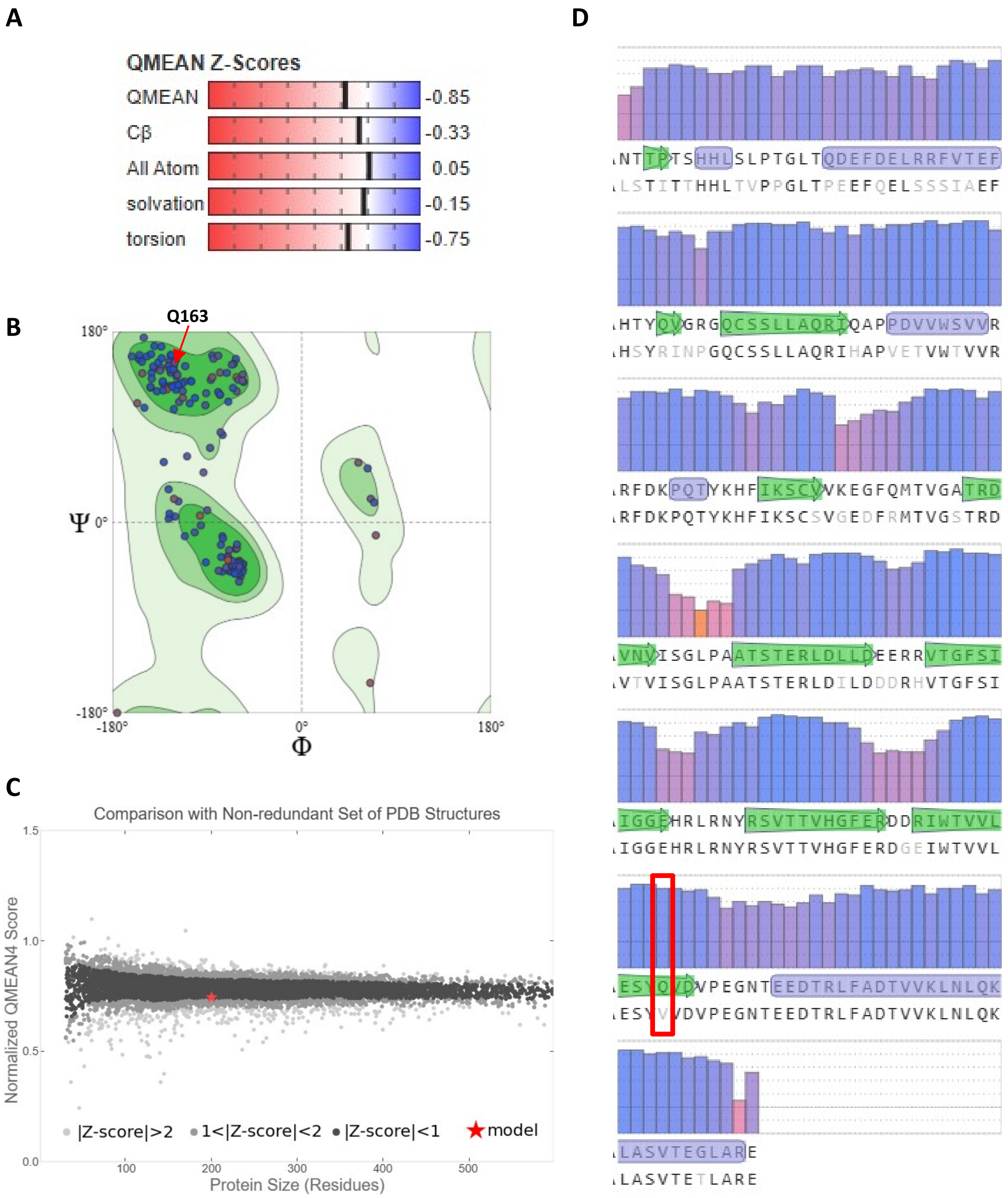

Supplemental figure 3. **Model quality parameters of HlPYL1b**. (A) QMEAN Z-scores, (B) Ramachandran plot, (C) a comparison of the normalized QMEAN4 score between HlPYL2 model and other PDB protein structures, and (D) the local QMEAN values were provided by swiss-model tool.

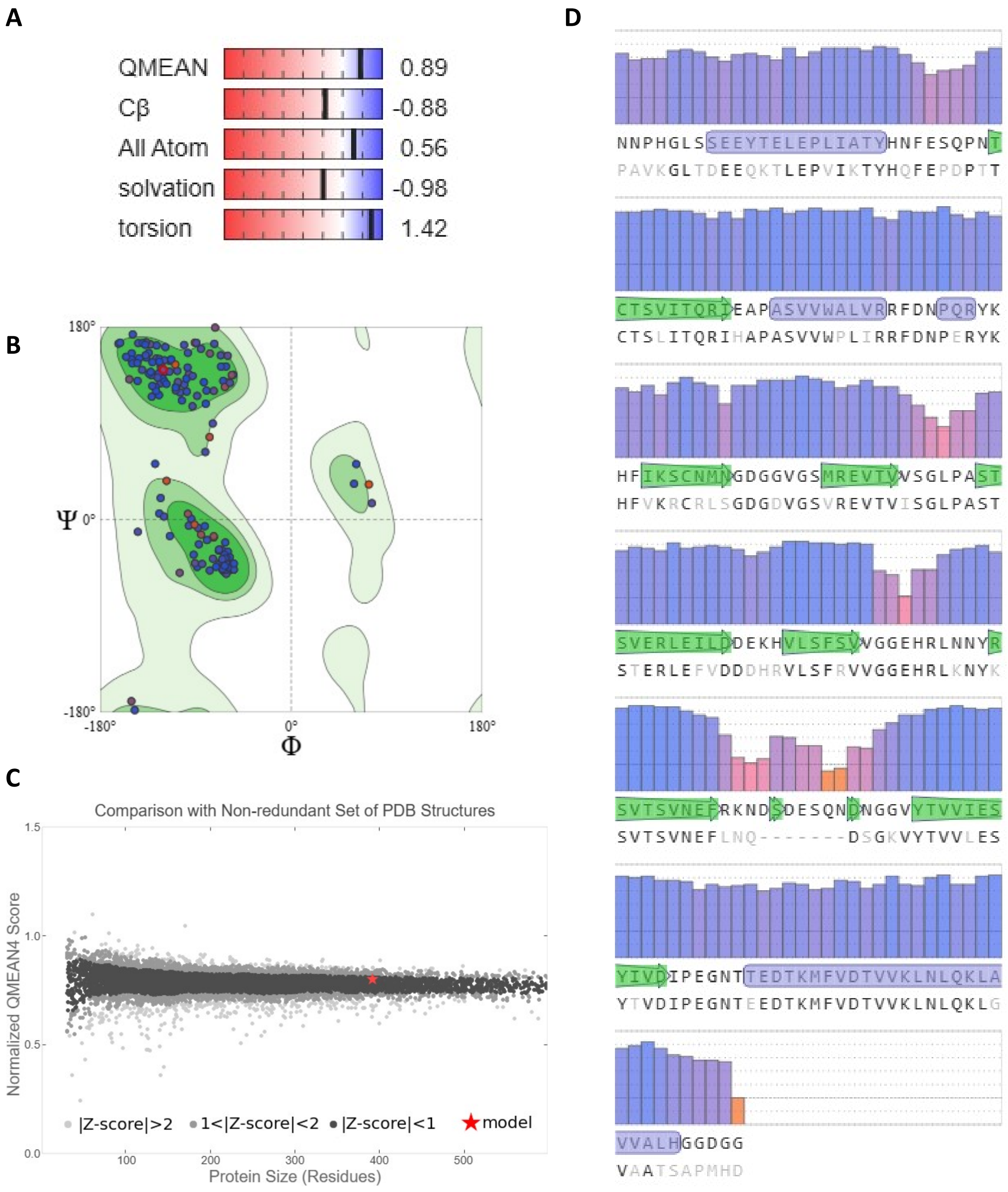

Supplemental figure 4. **Model quality parameters of HlPYL2**. (A) QMEAN Z-scores, (B) Ramachandran plot, (C) a comparison of the normalized QMEAN4 score between HlPYL2 model and other PDB protein structures, and (D) the local QMEAN values were provided by swiss-model tool.
